# Dietary intake in patients with ileal pouch-anal anastomosis: a cross-sectional comparative study

**DOI:** 10.1093/crocol/otag104

**Published:** 2026-09-24

**Authors:** Emre Kiliç, Ole Thorlacius-Ussing, Sabrina Just Kousgaard

**Affiliations:** Department of Gastrointestinal Surgery, Aalborg University Hospital, Aalborg, Denmark; Department of Gastrointestinal Surgery, Aalborg University Hospital, Aalborg, Denmark; Department of Clinical Medicine, Aalborg University, Aalborg, Denmark; Department of Gastrointestinal Surgery, Aalborg University Hospital, Aalborg, Denmark; Department of Gastroenterology & Hepatology, Aalborg University Hospital, Aalborg, Denmark

**Keywords:** inflammatory bowel disease, ulcerative colitis, ileal pouch-anal anastomosis, chronic pouchitis, diet

## Abstract

**Background:**

Ileal pouch-anal anastomosis (IPAA) is associated with good long-term outcomes, yet pouchitis remains a common complication. Although antibiotics are first-line therapy, recurrent disease often leads to repeated use with declining efficacy. Dietary interventions have shown promise, but evidence and consensus remain limited. The aim of this study was to compare dietary intake between IPAA patients with chronic pouchitis, IPAA patients with a normally functioning pouch, and healthy controls.

**Methods:**

This cross-sectional study recruited participants between November 2017 and October 2022. Participants were divided into 3 groups: patients with chronic pouchitis, patients with a normally functioning pouch, and healthy controls with an intact colon. Dietary intake over the previous 12 months was assessed using a food frequency questionnaire. Foods were categorized into groups and reported as servings per week.

**Results:**

The study included 30 participants with chronic pouchitis, 30 with a normally functioning pouch, and 82 healthy controls. Compared with the normal pouch function group, participants in the chronic pouchitis group consumed significantly more refined grains (mean difference [MD] 3.0; *P* = .045) and chocolate and candy (MD 2.5; *P* = .011). Compared with healthy controls, the chronic pouchitis group had a higher intake of sweets (MD 5.5; *P* = .001), refined grains (MD 3.8; *P* = .006), crispbread (MD 5.0; *P* < .001), and chocolate and candy (MD 2.6; *P* = .007).

**Conclusion:**

Dietary intake was generally similar between groups, but patients with chronic pouchitis reported higher consumption of starchy and sugar-rich foods. This dietary pattern may be associated with pouchitis, highlighting the need for interventional studies to assess causality.

## Graphical Abstract

**Figure:**
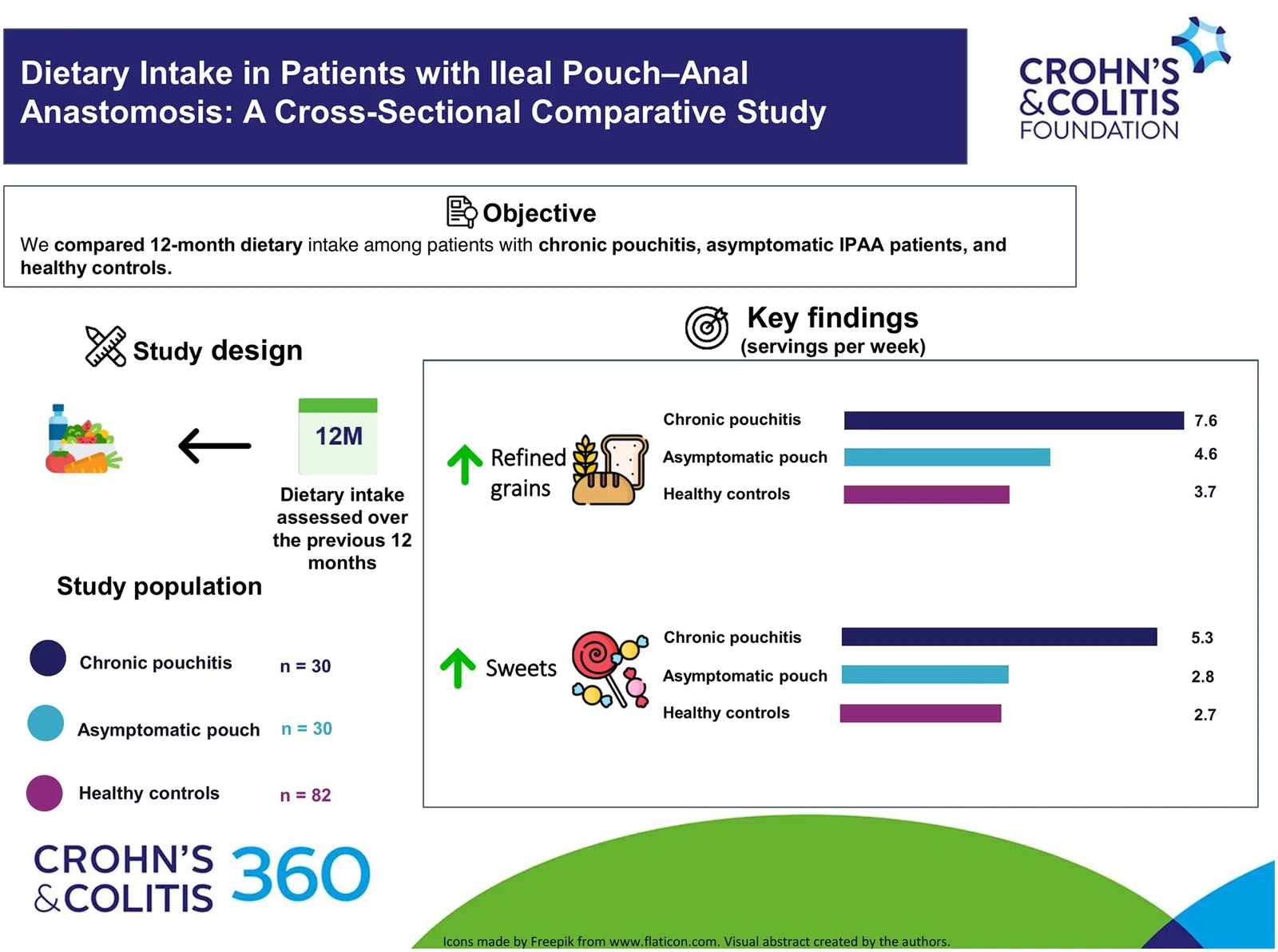

## Introduction

Restorative proctocolectomy with ileal pouch-anal anastomosis (IPAA) is the preferred surgical treatment for patients with ulcerative colitis (UC) refractory to medical therapy. The procedure usually involves the construction of a double-stapled, J-shaped pouch. Long-term results following IPAA have generally been favorable, with patients reporting good functional outcomes and improved quality of life.^1^^,^^2^ While IPAA surgery can be effective, pouch function varies, and postoperative complications are frequently reported. Pouchitis is one of the most common complications, with an incidence ranging from 11% to 61%.^3^ The cause of pouchitis is complex and influenced by multiple factors, including mucosal inflammation resulting from gut dysbiosis and an associated increase in pro-inflammatory cytokines.^4^

The first-line treatment for pouchitis is a 2-4-week course of antibiotics, typically ciprofloxacin and/or metronidazole.^5^^,^^6^ However, frequent relapses may lead to chronic antibiotic-dependent pouchitis or progress to chronic antibiotic-refractory pouchitis, for which biologic treatment may be necessary.^5^^,^^6^

In recent years, alternative approaches have emerged that aim to restore gut health by modulating intestinal function and microbial balance. Prebiotic and probiotic supplements containing strains of *Lactobacillus* and/or *Bifidobacterium* have been shown to help prevent pouchitis and reduce disease activity, although results have been inconsistent.^4^^,^^7^^,^^8^ Another suggested treatment is fecal microbiota transplantation (FMT). FMT is an effective treatment of recurrent *Clostridioides difficile* infection (rCDI), but evidence supporting its effectiveness in pouchitis remains limited.^8^

A recent survey found that nearly all patients with IPAA attempt to manage symptoms by adjusting their dietary intake.^9^ Therefore, there is increasing interest in dietary strategies to improve symptoms. Dietary patterns such as the *Mediterranean diet (MD)*^10^ are thought to stimulate the growth of beneficial bacteria and the production of short-chain fatty acids. The MD has been associated with a reduced incidence of pouchitis and symptom relief. Other diets, such as the *Crohn’s disease exclusion diet (CDED)*,^11^ are more comprehensive and focus on combining enteral nutritional formulas with the avoidance of certain food groups. This diet has been shown to improve remission rates in pouchitis, decrease PDAI-score, and reduce stool frequency.^4^^,^^8^^,^^12^ However, the relationship between diet and pouchitis remains difficult to interpret, as dietary intake may both influence pouch inflammation and be altered because of pouch-related symptoms.^9^ Research in this field remains limited, and existing studies are constrained by small sample sizes and heterogeneous study designs. The latest guidelines from the American Gastroenterological Association emphasize the limited knowledge regarding the impact of diet on the disease course of pouchitis.^5^ To our knowledge, formal evidence-based dietary guidelines have not yet been established. The present study aims to characterize and compare dietary intake patterns among patients with chronic pouchitis, a normally functioning pouch, and healthy controls, thereby contributing to the growing body of evidence needed to develop future dietary guidelines for patients with IPAA.

## Methods

### Study design

This study was designed as a cross-sectional study comparing dietary intake over the past 12 months using a validated food frequency questionnaire (FFQ) across 3 participant groups: patients with chronic pouchitis (CP), patients with a normally functioning pouch (NP), and healthy controls (HC).

### Setting

Participants were recruited between November 2017 and October 2022. Participants with CP were identified through the MicroPouch randomized controlled trial.^13^ Participants with an NP were enrolled through the Department of Gastrointestinal Surgery, Aalborg University Hospital, Aalborg, Denmark. Recruitment was carried out by telephone or during scheduled follow-up visits. HC were recruited via hospital announcements at Aalborg University Hospital.

### Participants

All participants were 18 years of age or older. All participants who had undergone IPAA surgery had UC as the underlying condition.

Participants with CP had undergone IPAA surgery at least 1 year prior to enrollment. CP was characterized by ≥3 episodes of relapsing pouchitis within a 12-month period or a single episode lasting ≥4 weeks that was not responsive to antibiotic treatment. Additionally, participants must have received ≥1 antibiotic treatment for pouchitis within the previous 3 months. Pouchitis was defined using the PDAI-score, with a total score of ≥7, including a clinical subscore ≥3 and endoscopic and histological subscores each ≥1. Participants with CP were excluded if they had any secondary causes of pouchitis or were immunocompromised.

Participants with an NP were defined by a clinical PDAI-score ≤2, absence of pouch dysfunction, stool frequency ≤8 stools per day, and no antibiotic use for pouchitis within 6 months prior to inclusion.

HC were individuals without known chronic conditions (except for allergies), with an intact colon and normal bowel movements (1-3 stools per day, Bristol Stool Form Scale types 3-4). Participants who had taken any medication and/or antibiotic treatment other than allergy and contraceptive medicine within the previous 6 months were excluded.

### Diet questionnaire

To assess dietary habits, a standardized and validated FFQ developed by the Technical University of Denmark Food Institute^14^ was used to record the participants’ dietary intake. The FFQ was validated in 2009,^15^ and was designed to efficiently capture participants’ dietary intake over the previous 12 months. Questions about food intake were formulated in accordance with national dietary guidelines. It was originally designed to monitor public health across Nordic countries. The FFQ included key food groups including grains, dairy, fruit, vegetables, meat, fish, snacks (eg, cakes, biscuits, candy, and chocolate), nuts, and beverages. Additional questions were added, including information about supplement intake, smoking status, and vegan diet. Supplements were defined as commercially available products such as vitamins, minerals, and probiotics.

### Statistics

Continuous variables were reported as mean ± standard deviation (SD), while categorical variables were reported as counts and percentages. Missing values were excluded from the calculations.

Education levels were categorized into 3 groups: primary school (≤10 years) and vocational education (1-2 years post-primary) were classified as lower education. High school/HF (2-3 years post-primary) and short education (1-2 years post-secondary) were classified as intermediate education. Medium education (3-4 years post-secondary) and higher education (≥5 years post-secondary) were classified as higher education.

Food items were grouped into matching food groups based on similarity to enhance clarity and facilitate interpretation. In total, food items were categorized into ten food groups (see Table S1). For each food group, participants reported their consumption frequency using categories ranging from daily to rarely, with corresponding frequency categories. These frequencies were then converted into standardized servings per week. Values reported as “<1” were assigned a value of 0.1, and “rarely” was defined as consuming the food item once every 4 months, to simplify the analysis. Q-Q plots of residuals were calculated for all food items and groups to assess normality prior to analysis. Due to non-normal distributions of the food group variables, differences between participant groups were evaluated using bootstrapped unpaired t-tests with 10 000 bootstrap replications. Results were reported as mean differences and 95% confidence intervals, and a *P*-value <.05 was considered statistically significant. A subgroup analysis was conducted for individual food item, to further evaluate each food item. Food items with either clinical or statistical significance were included in the Results section.

All statistical analysis was performed using StataCorp. 2023. *Stata Statistical Software: Release 18*. College Station, TX: StataCorp LLC.

### Ethics

The study was conducted in accordance with Good Clinical Practice guidelines and the principles outlined in the revised Declaration of Helsinki. Written informed consent was obtained from all participants prior to enrollment, and they retained the right to withdraw consent at any time during the study. Ethical approval was granted by the Regional Research Ethics Committee of Northern Jutland, Denmark (project numbers N-20150021 and N-20180013).

## Results

### Participant characteristics

A total of 142 participants were included in the study. Participants were categorized into 3 groups: 30 participants with CP, 30 participants with NP, and 82 HC.

The characteristics of the participants are presented in Table 1. Significant differences were observed in sex, age, and education level between the CP and NP groups. The mean clinical pouchitis disease activity index (cPDAI) score was higher in the CP group (3.8 ± 0.8) than in the NP group (1.1 ± 0.9). Mean stool frequency differed across all groups with the highest frequency observed in the CP group (12.1 ± 3.7), followed by the NP group (5.7 ± 1.4), while the HC group having the lowest frequency (1.6 ± 0.6). All other baseline demographics were comparable across the 3 groups.

**Table 1:** Participant characteristics by study group.

	Chronic pouchitis (*n* = 30)	Normal pouch function (*n* = 30)	Healthy controls (*n* = 82)
**Sex, female, frequency (%)**	20 (67.0)	10 (33.0)	41 (50.0)
**Age, years, mean (SD)**	49.1 (13.4)	58.6 (10.5)	47.8 (14.3)
**BMI, mean (SD)**	25.2 (4.5)	25.5 (3.2)	24.5 (3.4)
**cPDAI score, mean (SD)**	3.8 (0.8)	1.1 (0.9)	–
**Daily stool frequency, mean (SD)**	12.1 (3.7)	5.7 (1.4)	1.6 (0.6)
**Education, frequency (%)**			
** Higher**	25 (82.8)	15 (50)	55 (67.1)
** Intermediate**	4 (13.8)	13 (43.3)	26 (31.7)
** Lower**	1 (3.4)	2 (6.7)	1 (1.2)
**Active smoker, frequency (%)**	3 (10.0)	3 (10.0)	5 (6.1)
**Supplements, frequency (%)**	12 (40.0)	12 (40.7)	31 (38.3)

Abbreviations: BMI, body mass index; cPDAI, clinical pouchitis disease activity index.

### Dietary habits

#### Differences in food consumption between the CP and NP groups

Food consumption was generally comparable across most food groups between the two groups, as shown in Table 2. The CP group consumed significantly more refined grains than the NP group (mean difference 3.0, 95% CI [0.1-5.9], *P* = .045). A non-significant trend toward higher consumption of crispbread (mean difference 3.1, 95% CI [−0.2 to 6.4], *P* = .069) and vegetables (mean difference 2.1, 95% CI [−0.4 to 4.6], *P* = .094) in the CP group. A higher consumption of diet soda was observed in the NP group (mean difference −3.8, 95% CI [−8.3 to 0.7], *P* = .094) compared with the CP group.

**Table 2:** Dietary comparison between chronic pouchitis and normal pouch function group.

Food groups (food items)	Chronic pouchitis	Normal pouch function	Mean difference (95% CI)	*P*-value
**Fruit, mean (SD)**	8.5 (6.0)	6.7 (4.8)	1.8 (−1.1 to 4.6)	.231
**Vegetables, mean (SD)**	9.3 (4.9)	7.2 (4.9)	2.1 (−0.4 to 4.6)	.094
**Dairy, mean (SD)**	14.8 (12.2)	14.1 (9.5)	0.7 (−4.8 to 6.2)	.804
**Meat, mean (SD)**	10.0 (7.1)	8.5 (4.1)	1.5 (−1.4 to 4.4)	.309
**Sweets, mean (SD)**	11.2 (9.0)	8.3 (6.9)	2.9 (−1.1 to 6.9)	.150
** Sweets (chocolate/candy), mean (SD)**	5.3 (4.9)	2.8 (2.0)	2.5 (0.6-4.4)	.011
**Diet soda, mean (SD)**	2.5 (5.9)	6.3 (10.7)	−3.8 (−8.3 to 0.7)	.094
**Whole grains, mean (SD)**	19.3 (14.6)	21.4 (13.8)	−2.1 (−9.3 to 5.0)	.558
**Refined grains, mean (SD)**	7.6 (6.9)	4.6 (4.1)	3.0 (0.1-5.9)	.045
**Crispbread, mean (SD)**	9.1 (6.9)	6.1 (5.6)	3.1 (−0.2 to 6.4)	.069
**Nuts, mean (SD)**	3.1 (3.8)	2.5 (3.1)	0.5 (−1.3 to 2.3)	.582

Values are presented as servings per week.

In the subgroup analysis of individual food items, consumption of chocolate and candy was significantly higher in the CP group compared with the NP group (mean difference 2.5, 95% CI [0.6-4.4], *P* = .011).

#### Differences in food consumption between the CP and HC groups

Overall, food consumption was similar between the two groups, as shown in Table 3. Participants with CP consumed significantly more sweets (mean difference 5.5, 95% CI [2.1-8.8], *P* = .001) and refined grains (mean difference 3.8, 95% CI [1.1-6.6], *P* = .006) compared with the HC group. Furthermore, a significantly higher consumption of crispbread (mean difference 5.0, 95% CI [2.2-7.9], *P* <.001) was observed in the CP group.

**Table 3:** Dietary comparison between chronic pouchitis and healthy controls group.

Food groups (food items)	Chronic pouchitis	Healthy control	Mean difference (95% CI)	*P*-value
**Fruit, mean (SD)**	8.5 (6.0)	6.9 (5.0)	1.6 (−0.9 to 4.1)	.221
**Vegetables, mean (SD)**	9.3 (4.9)	10.0 (5.9)	−0.7 (−2.8 to 1.5)	.549
**Dairy, mean (SD)**	14.8 (12.2)	11.5 (9.2)	3.3 (−1.5 to 8.0)	.175
** Dairy (full-fat cheese), mean (SD)**	5.0 (5.5)	3.2 (2.9)	1.8 (−0.3 to 3.9)	.087
**Meat, mean (SD)**	10.0 (7.1)	10.6 (10.0)	−0.6 (−4.0 to 2.7)	.709
**Sweets, mean (SD)**	11.2 (9.0)	5.7 (4.9)	5.5 (2.1-8.8)	.001
** Sweets (chocolate/candy), mean (SD)**	5.3 (4.9)	2.7 (2.8)	2.6 (0.7-4.5)	.007
** Sweets (regular soda), mean (SD)**	3.5 (7.2)	1.2 (2.9)	2.3 (−0.4-5.0)	.090
**Diet soda, mean (SD)**	2.5 (5.9)	2.7 (4.0)	−0.2 (−2.5 to 2.1)	.868
**Whole grains, mean (SD)**	19.3 (14.6)	21.8 (16.0)	−2.5 (−8.7 to 3.7)	.428
**Refined grains, mean (SD)**	7.6 (6.9)	3.7 (5.3)	3.8 (1.1-6.6)	.006
**Crispbread, mean (SD)**	9.1 (6.9)	4.1 (5.3)	5.0 (2.2-7.9)	<.001
**Nuts, mean (SD)**	3.1 (3.8)	3.4 (5.6)	−0.4 (−2.3 to 1.5)	.686

Values are presented as servings per week.

In the subgroup analysis, consumption of chocolate and candy was significantly higher in the CP group compared with the HC group (mean difference 2.6, 95% CI [0.7-4.5], *P* = .007). A trend toward higher consumption of full-fat cheese (mean difference 1.8, 95% CI [−0.3 to 3.9], *P* = .087) and regular soda (mean difference 2.3, 95% CI [−0.4-5.0], *P* = .090) was observed in the CP group compared with the HC group.

## Discussion

In this cross-sectional study, our aim was to compare dietary intake among IPAA patients with CP to patients with NP, and HCs. We observed a statistically significantly higher intake of refined grains and chocolate/candy in the CP group. We also observed a trend toward higher consumption of crispbread and vegetables compared with the NP group. In contrast, the NP group tended to consume greater amounts of diet soda. When comparing the CP group with the HC group, the intake of sweets, chocolate/candy, refined grains, and crispbread was significantly higher in the CP group. A trend toward higher consumption of regular soda and full-fat cheese was also observed in the CP group compared with the HC group.

Overall, the CP group tended to consume greater amounts of carbohydrate-rich foods, particularly those high in starch and simple sugars. Foods such as refined grains and rice are often recommended for IPAA patients who experience active pouchitis, as they may help relieve symptoms, especially stool output and bloating.^12^^,^^16^ However, while starch may relieve symptoms, Goren et al. found that higher total starch consumption was associated with elevated anti-glycan antibodies AMCA and ACCA.^17^ These antibodies are associated with dysbiosis of the microbiome, including higher *Candida albicans* abundance, and may indirectly contribute to pouch inflammation. Refined grain products are rich in starch, for example commercially available packaged white bread, which undergo extensive processing to improve shelf life, texture, and palatability, often resulting in ultra-processed foods.^16^ Narula et al. observed that higher consumption of ultra-processed grains increased the risk of developing inflammatory bowel disease, whereas fresh bread and rice were associated with a reduced risk.^18^ Although pouchitis was not examined specifically, the findings suggest that such foods could increase the risk of pouchitis by increasing intestinal inflammation. Therefore, patients with active pouchitis should not necessarily be advised to avoid starch as a broad category. Instead, dietary guidance should distinguish between refined or ultra-processed starch-rich foods and less processed carbohydrate sources that provide dietary fiber or resistant starch, while also considering individual tolerance, stool frequency, and bloating.

Other food groups consumed more frequently in the CP group were vegetables and sweets. Certain vegetables (eg, onions and mushrooms) and sweets are rich in fermentable carbohydrates and may be poorly tolerated by some IPAA patients because of their high FODMAP content.^19^ Previous studies have found strong associations between high FODMAP foods and increased stool frequency, loose stools, bloating, and urgency in patients with IPAA.^16^^,^^20^ In a pilot study by Croagh et al., stool frequency improved significantly (8 to 4 stools/day) after a low FODMAP diet in IPAA patients without pouchitis, but no improvement in pouchitis was seen in the active pouchitis group.^21^ Hence, a low FODMAP approach may be useful for selected IPAA patients with increased stool frequency, loose stool, or bloating. However, it should not be interpreted as a restriction of fruits, vegetables, legumes, or whole grains. Rather, it should be implemented as a targeted and preferably dietitian guidance, with reintroduction and individual tolerance testing, to preserve nutrient-dense plant foods and dietary fiber if tolerated. This is particularly important because higher fruit intake, and potentially broader intake of fiber-rich plant foods, has been associated with greater microbial diversity and lower risk of pouchitis.^4^ Notably, some high FODMAP foods, particularly fruits, appear protective for patients with pouchitis. Godny et al. found that consuming ≥ 1.5 fruit servings/day reduced the risk of pouchitis.^22^ Similarly, Ianco et al. reported that patients with a normal pouch function consumed more fruit than those with pouchitis (3.6 servings/day vs 1.8 servings/day).^23^ This may be due to the high antioxidant and micronutrient content, and their ability to promote microbial diversity, which may make the microbiome more resilient to dysbiosis.^4^ The low intake of fruits and vegetables across all groups observed in our study suggests a clinically relevant deviation from dietary recommendations and a potential target for dietary modification.

In contrast to a low FODMAP diet, the Monash Pouch Diet (MPD) was designed to modify underlying disease mechanisms by increasing foods for beneficial fermentation (eg, oligosaccharides and resistant starch). However, in a double-blind RCT, all MPD participants experienced worsening symptoms, leading to early trial termination.^24^ This reflects a common challenge across many dietary interventions. Certain dietary interventions become overly strict and non-personalized, resulting in poor tolerability and high variability in effectiveness among patients with IPAA. Therefore, patients may have difficulty exploring alternative dietary approaches beyond those they currently find beneficial.^20^

To our knowledge, the MD and the CDED are among the most promising well-studied dietary approaches for pouch health. A cohort study of 153 patients with IPAA showed that higher adherence to the MD was associated with lower levels of inflammatory markers and a reduced risk of pouchitis onset.^16^^,^^25^ The CDED has also shown encouraging results. In a pilot study by Isakov et al., 67% of patients achieved remission in 6 weeks and 80% maintained remission at week 12, with almost half remaining in remission at the end of the 24-week trial.^26^ Stool frequency also improved (from 12.5 to 7 stools/day), and clinical PDAI scores decreased (from 8 to 5). The higher intake of refined grains and sugary foods observed in patients with CP in this study contrasts with the principles of the MD and CDED, which emphasize minimally processed foods and reduced intake of refined carbohydrates. However, further studies are needed to confirm the long-term effectiveness and feasibility of these dietary approaches in pouchitis. The MD is an inclusive dietary pattern that emphasizes nutrient-dense whole foods while limiting highly processed foods. Still, sustained adherence may be challenging if it requires substantial changes to patients’ habitual food choices. The CDED may pose a greater adherence burden because it is more structured and excludes or limits selected foods.

This study has several strengths. The inclusion criteria were carefully defined for each group, allowing for a clear distinction and minimizing the risk of overlap between the groups. Although this study included only 60 participants with IPAA and 82 healthy controls, the sample size is comparable to that of similar studies in this field. It is also important to emphasize that only IPAA patients with UC were included, making the groups more comparable.

One of the limitations of this study relates to its cross-sectional design, which only allows associations to be identified. Thus, even if certain foods, such as refined grains and sweets, were consumed in higher amounts by individuals with pouchitis, it is not possible to exclude reverse causation. An additional limitation relates to data collection. Because dietary intake over the previous 12 months was assessed at a single time point, recall bias cannot be excluded. Furthermore, disease status may have influenced participants’ ability to recall, as individuals with CP may be more aware of diet-related factors due to their condition. Lastly, the lack of validated dietary quality indices limits the interpretation of the overall dietary quality observed in patients with pouchitis.

The FFQ assessed intake as servings per week, whereas many studies use servings per day. Although conversion was possible, it was not performed because the low reported frequencies for some foods would have reduced interpretability. In addition, the subjective nature of “servings” and the FFQ definitions may have limited the accuracy of intake estimates. Finally, the statistical analysis required multiple testing, and the bootstrapped t-test is a robust method for comparing means, multiple comparisons still increase the risk of false-positive results. A Bonferroni correction was calculated (adjusted *P* ≈ .0025) but not applied, as its conservativeness would increase the risk of type II error given the small sample size and limited statistical power.

Future studies would benefit from using food diaries to reduce recall bias and allow dietary changes to be tracked. Moreover, dietary studies should consider patients’ personal dietary experiences. Rather than expecting patients to abandon their habitual eating patterns in favor of a standardized diet, future interventions may benefit from an individualized, dietitian-guided dietary approach based on principles from the MD and CDED. This would include reduced intake of refined carbohydrates, sugary foods, and highly processed foods, while ensuring tolerability, adherence, and nutritional adequacy. Finally, incorporating validated dietary quality indices may provide further insight into dietary patterns associated with pouchitis and help identify clinically relevant dietary targets that could inform nutritional guidance and future intervention studies.

## Conclusion

This study found overall similar dietary intake among patients with CP, patients with an NP, and HCs. Nevertheless, patients with CP consumed significantly higher amounts of refined grains, crispbread, and sugary snacks, as well as a tendency toward higher consumption of vegetables, full-fat cheese, and sugary beverages. Some of these foods may be associated with a higher risk of pouchitis; however, dietary recommendations cannot be confidently derived solely from the present findings. These findings may, however, be relevant in future interventional studies in patients with IPAA.

## Supplementary Material

otag104_Supplementary_Data

## Data Availability

The data supporting the findings of this study are available from the corresponding author upon request.
