# Supplementary material for: Dietary intake in patients with ileal pouch-anal anastomosis: a cross-sectional comparative study
Source: Crohns Colitis 360. 2026 Sep 24;8(4):otag104. doi: 10.1093/crocol/otag104 (PMC13680099; doi:10.1093/crocol/otag104)
Supplement: Supplementary file 1 [file otag104_supplementary_data.zip › Supplementary Data.docx]

# Supplementary Data

**Table 1. Food Group Classifications**

| **Food Group** | **Included Items** |
| --- | --- |
| Fruit | Fruit and berries |
| Vegetables | Vegetables, legumes, root vegetables, raw vegetables/plant-based foods |
| Dairy | Full-fat cheese, fermented dairy products, milk |
| Meat | Fish, meat, sausages |
| Sugar | Chocolate/biscuits, cake/biscuits/pie, regular soft drinks, energy drinks |
| Sugar-free drinks | Diet/sugar-free beverages |
| Whole-grain bread | Rye bread, whole-grain bread |
| White bread | White bread/French bread |
| Nuts | Nuts |
